# Second‐line lurbinectedin as a new treatment option for small‐cell lung cancer: Preliminary results in real‐clinical practice

**DOI:** 10.1111/1759-7714.14464

**Published:** 2022-06-17

**Authors:** Anne‐Claire Toublanc, Marina Guecamburu, Rémi Veillon, Pietro Rosellini, Pierre‐Olivier Girodet, Maeva Zysman

**Affiliations:** ^1^ Pulmonary Department, Pôle Cardio‐thoracique CHU de Bordeaux Bordeaux France; ^2^ INSERM, Centre de Recherche Cardio‐Thoracique de Bordeaux Bordeaux France; ^3^ Centre de Recherche Cardio‐thoracique de Bordeaux Univ‐Bordeaux Bordeaux France

**Keywords:** lurbinectedin, safety profile, salvage chemotherapy, small cell lung cancer

## Abstract

**Introduction:**

Few strategies exist for treatment of patients with small‐cell lung cancer (SCLC) extended‐stage after failure of first‐line platinum‐based chemotherapy. Lurbinectedin is a novel RNA‐polymerase‐II inhibitor investigated as a second‐line therapy for SCLC. However, its efficacy and safety profile in real clinical practice remain to be determined.

**Objective:**

To determine the efficacy and safety of lurbinectedin in real‐life among patients with SCLC previously treated with first‐line platinum‐based chemotherapy.

**Methods:**

We retrospectively evaluated patients who received at least one dose of lurbinectedin (3.2 mg/m^2^) between March 2020 and November 2021, in the pulmonary department of Bordeaux University Hospital. Endpoints were time to treatment discontinuation, progression‐free survival, overall survival, and safety profile.

**Results:**

Thirteen patients were included. The median age was 60 years (range: 42–77), seven (54%) were females, nine (69%) having a performance status of 0–1. Lurbinectedin was given as second‐line treatment before platinum rechallenge in four (31%) patients. After a mean follow‐up of 4.1 months, the objective response rate (ORR) was 17%. The median time to treatment discontinuation (TTD) was 2.3 months (interquartile range [IQR], 1.2–3.6). The median progression‐free survival (PFS) and overall survival (OS) were, respectively, 1.9 (IQR, 0.1.8) and 4.1 (IQR, 2.0–3.5) months. No significant difference regarding TTD, PFS or OS was found in the two groups according to treatment history or according to chemotherapy‐free intervall (CMI) 〈1 or 〉1 month. The most common adverse events (AEs) were asthenia, nausea, and anemia in nine (70%) patients. Grade 3 AEs were reported, fatigue, vomiting, nausea, anorexia, and neutropenia.

**Conclusions:**

Lurbinectedin in real clinical practice could have had a lower efficacy than in phase II trial, but a better hematological and bioclinical tolerance than previously reported. Early relapse after platinum‐based chemotherapy seems to have a lower response to lurbinectedin.

## Graphical abstract

We retrospectively evaluated patients with extended stage small‐cell lung cancer (ES‐SCLC) who received at least one dose of lurbinectedin between March 2020 and November 2021, in the pulmonary department of Bordeaux University Hospital. The study demonstrated that lurbinectedin in real clinical practice could have had a lower efficacy than in phase II trial, but a better hematological and bioclinical tolerance than previously reported. Refractory relapse (chemotherapy‐free interval [CMI] <1 month) after platinum‐based chemotherapy seems to have a lower response to lurbinectedin.

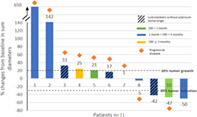

## INTRODUCTION

Small‐cell lung cancers (SCLCs) represent 13%–15% of all lung cancer.^1^ Because of their high mutation rate and tumor mutational burden, it is characterized by its aggressivity and rapid cellular proliferation. An initial high chemosensitivity is followed by a quick resistant emergency. For extensive stage SCLC (ES‐SCLC), combined chemotherapy (platinum‐etoposide) and immunotherapy (atezolizumab or durvalumab during and after chemotherapy) has become the new standard front‐line treatment, with modest improvement in overall survival (OS).^2^, ^3^

Few options exist for relapsed SCLC after the first‐line therapy. For years, topotecan was the only approved drug for second‐line treatment of patients with sensitive relapse.^4^ However, its use should be carefully considered given its hematological toxicities and its relatively low clinical benefit. Topotecan, lurbinectedin, and clinical trials are reasonable treatment options in refractory relapse.^1^, ^2^, ^3^, ^4^ Platinum‐rechallenge,^5^ topotecan, or lurbinectedin can be considered for sensitive relapse.

Lurbinectedin, which is a selective inhibitor of oncogenic transcription that binds preferentially to guanine located in the GC‐rich regulatory areas of deoxyribonucleic acid (DNA) gene promoters, is a new option as second‐line therapy for SCLC.

A single‐arm, open‐label, phase II basket trial^1^ showed encouraging results in patients treated with lurbinectedin as second line. Better OS, progression‐free survival (PFS), and better safety profile have been reported than with topotecan. OS, PFS, and overall response rate (ORR), of lurbinectedin were reported to be, respectively, 9.3, 3.5 months and 35.2%, whereas OS, PFS, and ORR of topotecan were 7.8, 4.2 months, and 16.9%, respectively. In terms of safety, topotecan caused treatment related death (TRD) because of hematologic toxicity in 2% of cases. As for lurbinectedin, no TRD was reported. Considering these results, topotecan seems a less attractive choice than lurbinectedin from the perspective of efficacy and safety.

The aim of this real‐clinical practice study was to evaluate the efficacy and safety profile of lurbinectedin following first‐line platinum‐based chemotherapy in patients with ES‐SCLC.

## MATERIALS AND METHODS

### Study population

We performed a monocentric retrospective study in the pulmonary department of University Hospital of Bordeaux. All 13 consecutive patients with ES‐SCLC, who received at least one administration of lurbinectedin, at a dose of 3.2 mg /m^2^, after first‐line platinum‐based chemotherapy, between March 1, 2020 and November 1, 2021 were included.

Between March 1, 2020 and November 1, 2021, 184 patients received first line platinum doublet chemotherapy for ES‐SCLC. Among the 184 patients, 70 patients had disease progression. Among 70 patients, 13 received lurbinectedin as second line therapy (Figure S1). We divided patients into two groups according to treatment history. Patients who had platinum doublet rechallenge therapy were classified into the “rechallenge group” and those who did not receive platinum doublet re‐challenge were classified as the “non‐re‐challenge group.” Patients who had a chemotherapy free interval (CFI) for more than 1 month was classified into “CFI >1 month,” and patients who had CFI <1 month was classified into “CFI 1 month.”

We used granulocyte colony‐stimulating factor (G‐CSF) as primary prophylaxis for febrile neutropenia.

### Data collection

Data on clinical and pathological features were extracted from the electronic medical record. We analyzed time to treatment discontinuation (TTD), PFS, OS, disease control rate (DCR), and ORR. We also evaluated the safety profile of lurbinectedin according to Common Terminology Criteria for Adverse Events v4.0 (NCI‐CTCAE).

TTD was defined as time from the first day of lurbinectedin administration to the treatment discontinuation or death; PFS was defined as time from the first day of lurbinectedin until progression or death; OS was defined as time from the first day of lurbinectedin to death or lost follow up; DCR was defined as the percentage of patients achieving a complete, partial, or stable disease and ORR corresponding to a complete or partial response (RECIST 1.1 criteria).^6^

### Statistical analysis and ethical considerations

The data were summarized by frequency and percentage for categorical variables and by median and range for continuous variables.

Qualitative variables were described with numbers and percentages, and quantitative variables with numbers of non‐missing data, median with first and third quartiles or range. Survival variables were described with survival probabilities and curves using Kaplan–Meier method. The survival endpoints were compared using a log‐rank test. A value of *p* ≤ 0.05 was considered statistically significant. All analyses were performed using Graph Pad Prism statistical software (GraphPad Software).

The study was conducted in accordance with French legislation and ethical codes. This work complies to the protection of personal health data and the protection of privacy with the framework of application provided for by article 65‐2 of the amended Data Protection Act and the general data protection regulations and was approved by an institutional review board and registered (no. CHUBX2021RE0113). The study was designed according to the STROBE guidelines.

## RESULTS

### Patient characteristics

A total of 13 patients were assessed. The median age at the first administration of lurbinectedin was 63 years (range: 42–77) (Table S1). Most of the patients were female (54%) and current smokers (92%). At lurbinectedin initiation, nine (69%) patients had performance status (PS) 0 to 1.

Three patients had chemotherapy‐free interval (CMI) less than one month, eight patients had CMI between one and three months and two had CMI more than three months. Four patients had lurbinectedin without platinum‐rechallenge. Three patients were ongoing treatment at the end of the study. Ten died because of disease progression.

Metastatic sites occured in liver in eight patients (62%), in lung in three (23%), in bones in three (23%), in central nervous system (CNS) in two (15%), in pleural and adrenal glands in one (8%) patient each one. Four (31%) patients received lurbinectedin as a second line and nine (69%) as third line or further treatment. The median CFI before lurbinectedin administration was 2.3 months (IQR, 1.9–3.3), corresponding to <1 month in three (23%) patients, between 1 and 3 months in eight (62%) patients and >3 months in two (15%) patients. Forty‐six treatment cycles in total were administered, with a median of three (IQR, 3–4) cycles per patient.

### Efficacy of lurbinectedin in real‐practice

The median follow‐up was 4.1 months. The median TTD was 1.7 months (IQR, 1.2–3.6). The investigator‐assessed mPFS was 1.9 months (IQR, 0.9–1.5) (Figure S2). The mOS was 4.1 months (IQR 0. 9–8.8). Three patients were censored because of ongoing treatment.

The ORR was 31% (*n* = 4). Among four patients who had partial response (Figure S3), two had lurbinectedin without platinum‐rechallenge, two had lurbinectedin with platinum‐rechallenge, one had CFI <1 month, and three had CFI more than 1 month.

DCR was 17% (Figure S3). All of them had lurbinectedin as second line of treatment; two patients had a partial response according to RECIST criteria. All other patients had a progressive disease.

### Efficacy according to treatment history and CFI

**FIGURE 1:**
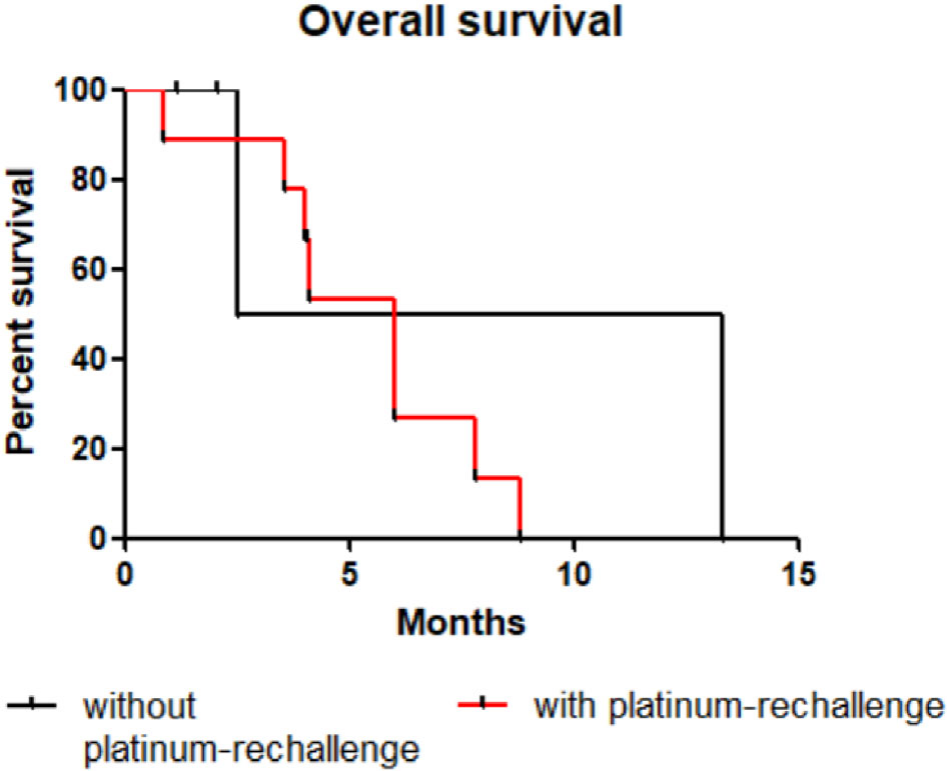
Kaplan–Meier overall survival curve for patients treated with lurbinectedin without or with platinum‐rechallenge. Difference overall survival was not relevant (*p* = 0.7).

**FIGURE 2:**
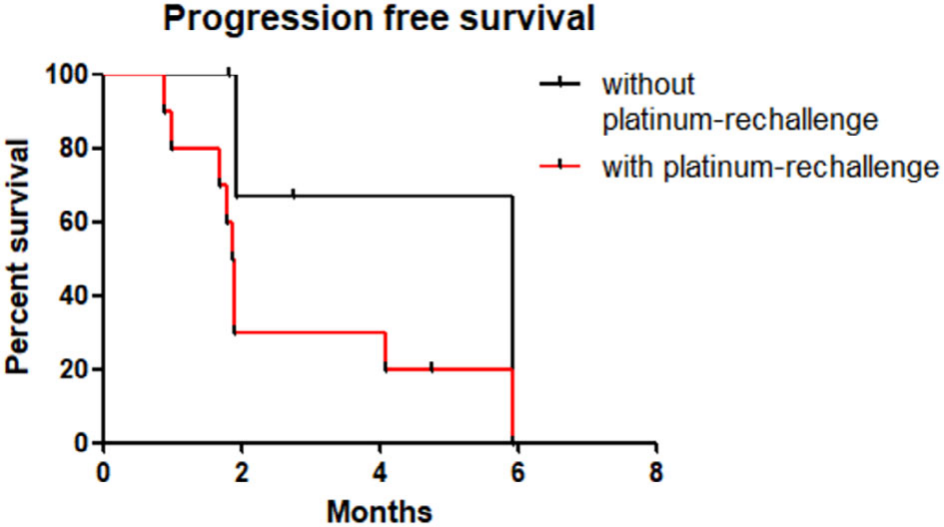
Kaplan–Meier progression‐free survival for patients treated with lurbinectedin without or with platinum‐rechallenge. Difference time to treatment discontinuation was not relevant (*p* = 0.07).

**FIGURE 3:**
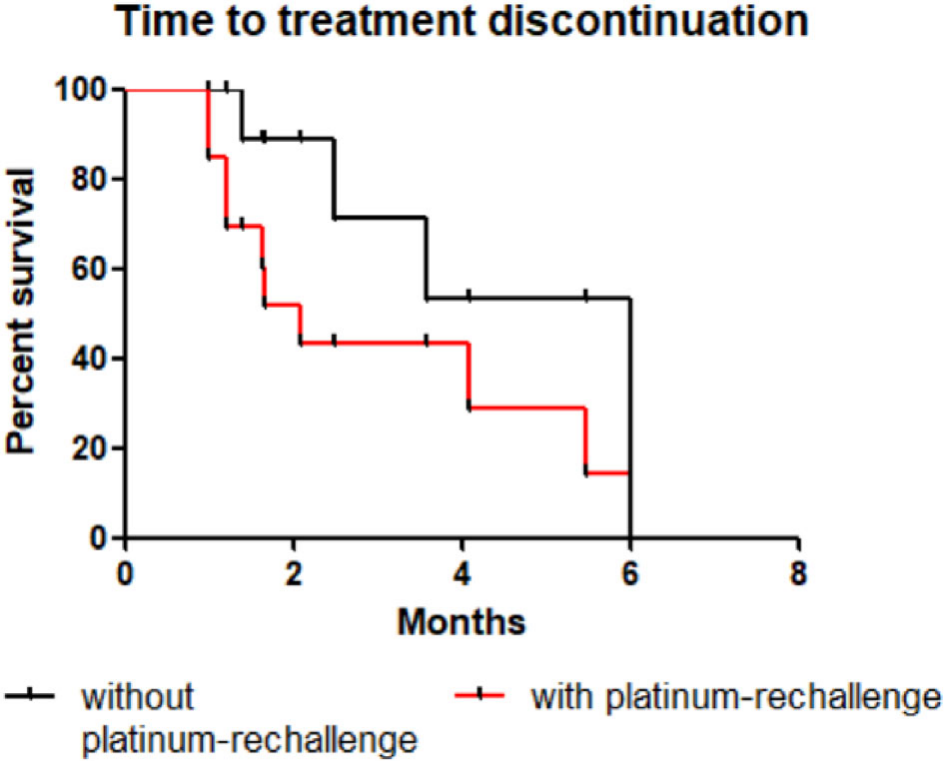
Kaplan–Meier time to treatment discontinuation for patients treated with lurbinectedin without or with platinum‐rechallenge. Difference time to treatment discontinuation was not relevant (*p* = 0.16).

No significant difference in OS, PFS and TTD was found according to platinum rechallenge (Figures 1, 2, 3). The median OS, PFS and TTD were respectively 7.9 months (IQR, 2.5–13.3), 5.9 months (IQR, 1.9–5.1) and 2.1 months (IQR, 1.9–4.7) in patients who had lurbinectedin without platinum‐rechallenge versus 6 months (IQR, 0.9–8.8), 1.9 months (IQR, 1.7–3.0) and 6 months (IQR, 1.2–3.8) in patients who had lurbinectedin after platinum‐rechallenge.

**FIGURE 4:**
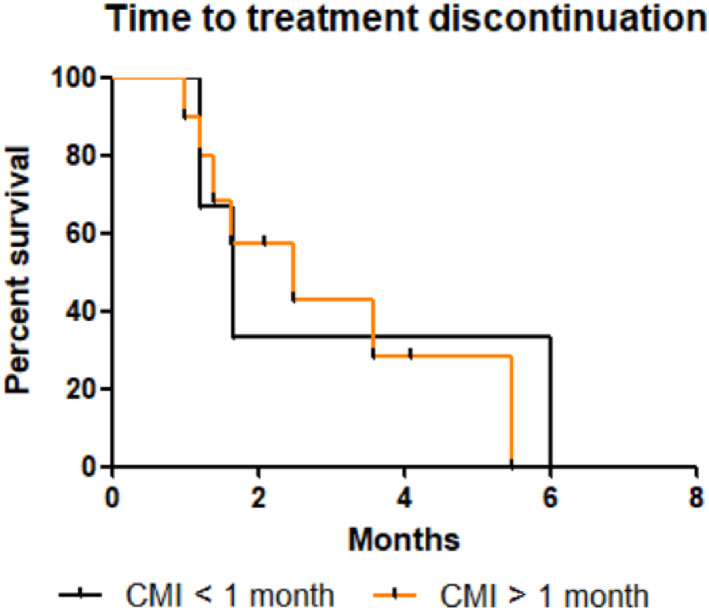
Kaplan–Meier time to treatment discontinuation depending on CMI. Difference time to treatment discontinuation was not relevant (*p* = 0.71). Abbreviation: CMI, chemotherapy‐free interval.

**FIGURE 5:**
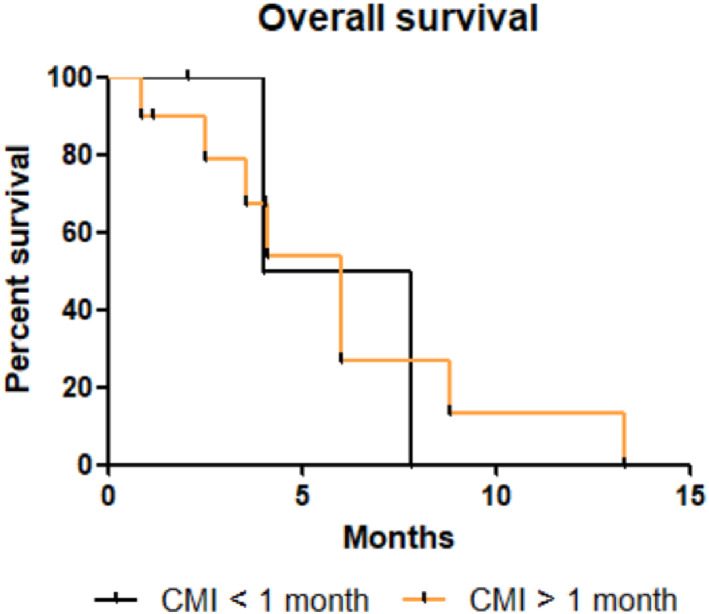
Kaplan–Meier overall survival curve depending on CMI. Difference overall survival was no relevant (*p* = 0.45). Abbreviation: CMI, chemotherapy‐free interval.

**FIGURE 6:**
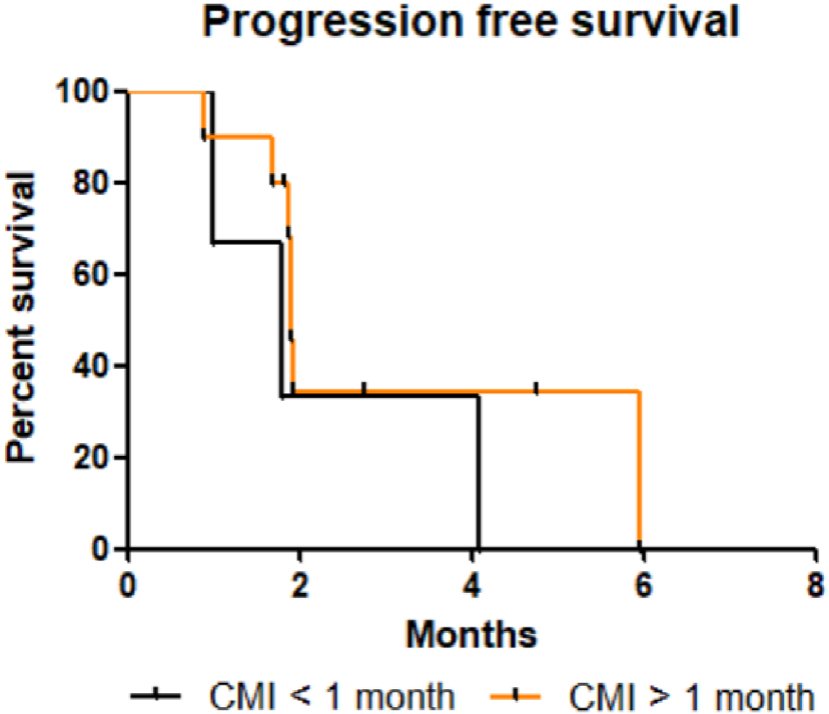
Kaplan–Meier progression‐free survival depending on CMI. Difference time to treatment discontinuation was no relevant (*p* = 0.3). Abbreviation: CMI, chemotherapy‐free interval.

No significant difference in OS, PFS and TTD was found according to CFI (Figures 4, 5, 6). The median OS, PFS and TTD were respectively 6 months (IQR, 5‐7.8), 1.8 months (IQR, 1.8‐2.8) and 1,7 months (IQR, 1,4‐6) in patients with CFI 〈1 month versus 4.1 months (IQR, 2.5‐7.4), 1.9 months (IQR, 1.4‐4.1) and 3.9 months (IQR, 1.2‐3.7) in patients with a CFI 〉1 month.

### Safety of lurbinectedin

All 13 treated patients were evaluated for safety (Table S1). In total, seven patients experienced hematological adverse events. One patient experienced grade 3 neutropenia. Anemia occurred in five (38%), all of whom were grade 1. Neutropenia occurred in one (8%) patient, who was classified as grade 3, because of febrile neutropenia.

One patient had gamma glutamyl transferase elevation grade 1, one patient had grade 2, and one patient had grade 3, without clinical consequence. One patient had grade 1 alkaline phosphatase elevation.

All of the 13 patients had at least one clinical symptom. Fatigue occurred in nine (69%) patients, one patient had grade 1, five patients had grade 2, and three patients had grade ≥3. Nausea was reported in eight (66%) patients, four patients had grade 1, three patients had grade 2, and one patient had grade ≥3. Anorexia and vomiting occurred in three patients, two patients had grade 1 and one patient had grade ≥3. One patient had diarrhea grade 2. Only one dose modification was reported (−25%) because of digestive toxicity with loss of 6% of weight in 1 month (grade 1).

No treatment‐related deaths occurred.

## DISCUSSION

Although modest improvements in survival have been made, especially in the metastatic setting with chemoimmunotherapy,^2^, ^3^ second‐line treatments in early relapsing SCLC remain challenging. Our study was the first retrospective study to evaluate the efficacy and safety profile of lurbinectedin in real‐life clinical practice.

In our study, lurbinectedin demonstrated lower efficacy in real‐life clinical practice than in pivotal trials. The median TTD, PFS, and OS were inferior to the results of phase II trial of lurbinectedin, respectively, 5.3 months, 35.2%, 3.5 months, and 9.3 months versus 1.7 months, 17%, 1.9 months, and 4.1 months in real‐life clinical practice.

Several hypotheses could be presented as reasons why efficacy of lurbinectedin was lower than in phase II trial. First, in our study, only two (15%) patients had CFI >3 months, whereas those patients with CFI >3 months are expected to respond favorably and durably to lurbinectedin.^1^ Second, in our study, patients with brain metastases were included, whereas those patients were excluded from the phase II trial of lurbinectedin.^1^ Third, in our study, lurbinectedin was administered as a second line in 31% as compared to 93% in the phase II trial. In our study, we would propose that lurbinectedin could be preferred in second line without platinum rechallenge than after the platinum rechallenge (hazard ratio = 3), but strategies need to be specifically evaluated in larger cohorts.

According to phase III trial of topotecan, 10% of febrile neutropenia were reported. Given this comparatively high incidence of febrile neutropenia, lurbinectedin could be comparable in terms of safety. In our study, only one case of febrile neutropenia was observed. This was much lower than previously published data.^1^ The lurbinectedin treatment regimen had lowered hematological and bioclinical side effects than expected with an acceptable and manageable safety profile, improved by a systematic primary prophylaxis by G‐CSF.

In our study, overall response did not differ between lurbinectedin (16%) than in phase III trial of topotecan (17%).^4^

For patients with relapse of SCLC, platinum‐rechallenge is advised in the National Comprehensive Cancer Network and the European Society of Medical Oncology^7^, ^8^ based on two studies: a retrospective one^5^ and a phase III randomized trial.^9^ The median PFS and median OR were both higher in the platinum rechallenge group^9^ than in the topotecan^4^ or lurbinectedin group. These results suggest that in case of delayed relapse, defined as a relapse occurring more than 6 months after the end of chemotherapy regimen, a rechallenge with first‐line platinum‐based should be prioritized. In case of sensitive relapse between 3 and 6 months, we would propose that lurbinectedin could be preferred; however, both strategies need to be specifically evaluated in larger cohorts.

This study has several important limitations. First, our study is a monocentric retrospective study analyzing small number of patients. Second, evaluation of tumor response was exerted by each attending physician, which means that evaluation could be quite arbitrary.

This study does not definitively address the optimal strategy to implement lurbinectedin administration after relapse. However, the strategy for treatment of patients with SCLC is slowly improving.

The lack of either a control or a recent historic control for SCLC also makes interpretation of the PFS and OS data challenging.

## CONCLUSION

Our study analyzed the efficacy and safety profile of lurbinectedin for pretreated SCLC in real‐life clinical practice. Our data suggest that lurbinectedin could become the standard second line treatment after ES‐SCLC progression if the chemotherapy free interval is <6 months. Studies with larger numbers of patients would help define the best indication for lurbinectedin after failure of first‐line platinum‐based chemotherapy in SCLC patients.

## CONFLICT OF INTEREST

No conflict of interest.

## Supporting information


Figure S1
Figure S2
Figure S3
Table S1
Table S2
