# Supplementary material for: Second‐line lurbinectedin as a new treatment option for small‐cell lung cancer: Preliminary results in real‐clinical practice
Source: Thorac Cancer. 2022 Jun 17;13(15):2248–52. doi: 10.1111/1759-7714.14464 (PMC9346176; doi:10.1111/1759-7714.14464)
Supplement: Supplementary file 1 — Figure S1 Figure S2 Figure S3 Table S1 Table S2 [file TCA-13-2248-s001.doc]

**Second-line lurbinectedin as a new treatment option for small-cell lung cancer: Preliminary results in real life practice**

AC Toublanc1, M. Guecamburu1, R. Veillon1, P. Rosellini1,3, PO Girodet1,2,3, M. Zysman1,2,3

1. CHU de Bordeaux, Pulmonary Department, Pôle Cardio-thoracique, CIC1401, Bordeaux, France.

2. Univ-Bordeaux, Centre de Recherche Cardio-thoracique de Bordeaux, U1045, Bordeaux, France.

3. INSERM, Centre de Recherche Cardio-Thoracique de Bordeaux, U1045, CIC 1401, Bordeaux, France.

SUPPLEMENTARY

**Figure S1**


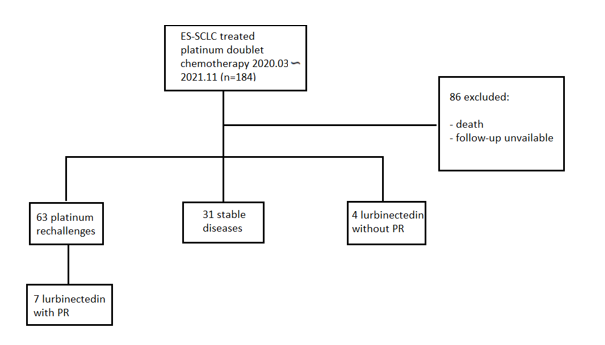


*Flow chart : ES-SCLC between March, 2020 and November, 2021, in the pulmonary department of Bordeaux University Hospital who received first-line platinum-based chemotherapy, between March, the 1^st^, 2020 and November, the 1^st^, 2021.*

**Table S1.**

| SUBJECTS | N=13 |
| --- | --- |
| Gender, n (%) |  |
| Female/Male | 7 (54)/ 6 (46) |
| Smoking history, n (%) |  |
| Never-smoker | 1(8) |
| Current or ex-smoker | 12(92) |
| Age in years, median (range) | 60 (42-77) |
| Performance Status, n (%) |  |
| 0 | 3 (23) |
| 1 | 6 (46) |
| 2 | 3 (23) |
| ≥3 | 0 |
| Missing | 1 (8) |
| Chemotherapy free interval, n (%) |  |
| < 1 month | 3 (23) |
| 1-3 months | 8 (61) |
| ≥ 3 months | 2 (15) |
| Time of lurbinectedin administration, n (%) |  |
| Without platinum-rechallenge | 4 (31) |
| With platinum-rechallenge | 9 (69) |
| Metastatic site, n (%) |  |
| Liver | 8 (62) |
| Lung | 3 (23) |
| Bones | 3 (23) |
| CNS | 2 (15) |
| Pleural | 1 (8) |
| Adrenal glands | 1 (8) |

*Clinical and pathological characteristics of patients before lurbinectedin administration.*


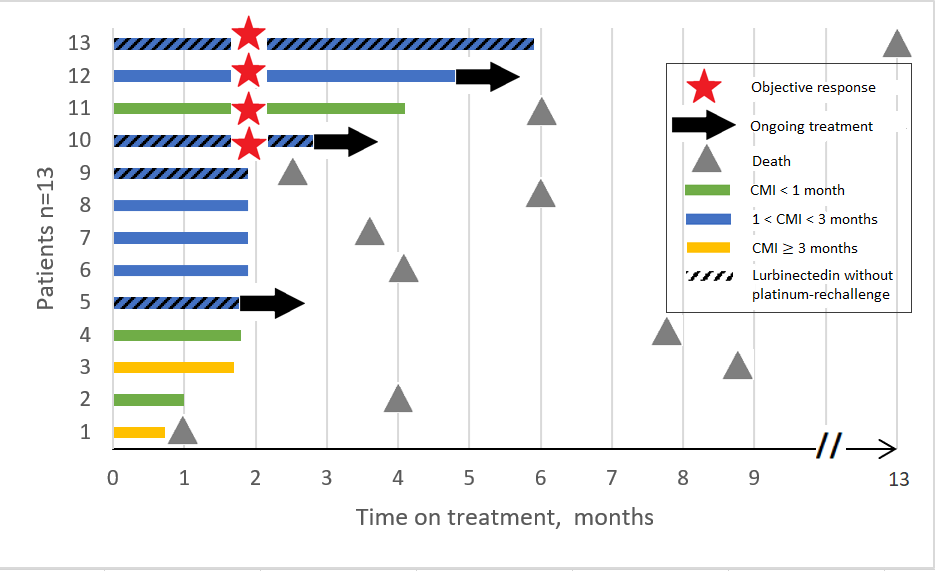
**Figure S2**

*Progression free survival. Each bar represents a patient with SCLC who received at least one dose of lurbinectedin (n=13). Green bar: CMI<1 month; blue bar:1month< CMI <3 months; yellow bar:CMI ≥ 3 months. Hatched bar: Lurbinectedin without platinum rechallenge*

*CMI : Chemotherapy free interval*

**Figure S3**

**
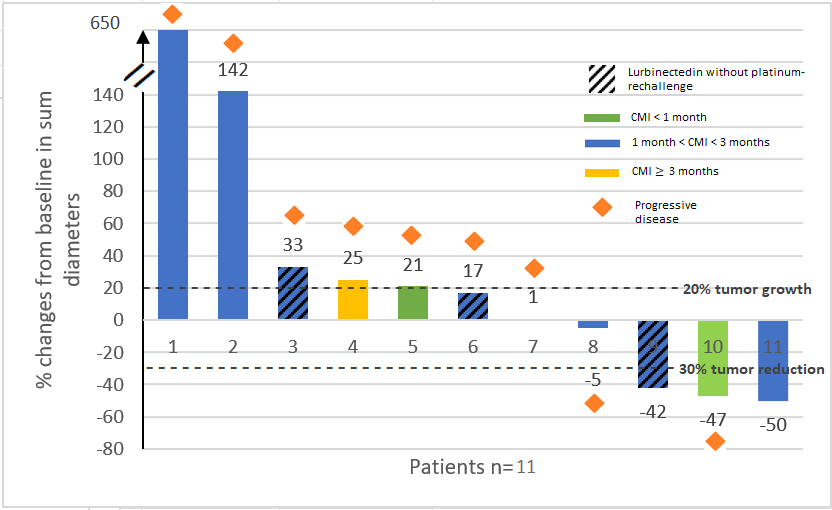
**

*Waterfall plot showing % change from baseline in sum of diameters after three cycles of lurbinectedin*, g*reen bar: CMI <1 month; blue bar : 1month < CMI <3 months; yellow bar : CMI ≥ 3 months. Hatched bar: Lurbinectedin without platinum rechallenge.*

*Two patients excluded because one died before scannographic reevaluation, and one just began lurbinectedin treatment.*

*CMI : Chemotherapy free interval*

**Table S2.**

|  | Grade 1 | Grade 2 | ≥Grade 3 |
| --- | --- | --- | --- |
| Haematological abnormalities |  |  |  |
| Anemia | 5 (38%) | 0 | 0 |
| Neutropenia | 0 | 0 | 1 (8%) |
| febrile neutropenia | 0 | 0 | 1 (8%) |
| Thrombopenia | 0 | 0 | 0 |
| Laboratory findings |  |  |  |
| gamma glutamyl transferase | 1(8%) | 1(8%) | 1(8%) |
| alkaline phosphatase | 1(8%) | 0 | 0 |
| Clinical symptoms |  |  |  |
| Fatigue | 1 (8%) | 5 (38%) | 3 (23%) |
| Nausea | 4 (31%) | 3 (23%) | 1 (8%) |
| Anorexia | 2 (15%) | 0 | 1 (8%) |
| Vomiting | 2 (15%) | 0 | 1 (8%) |
| Diarrhea | 0 | 1 (8%) | 0 |
| weight loss | 1 (8%) | 0 | 0 |

*Most common NCI-CTCAE laboratory abnormalities and treatment-related adverse events. Data are n (%) of patients. NCI-CTCAE=National Cancer Institute Common Terminology Criteria for Adverse Events version 4.0*.
